# What factors influence parents’ perception of the quality of life of children and adolescents with neurocardiogenic syncope?

**DOI:** 10.1186/s12955-016-0476-9

**Published:** 2016-05-17

**Authors:** Teresa Grimaldi Capitello, Caterina Fiorilli, Silvia Placidi, Roberta Vallone, Fabrizio Drago, Simonetta Gentile

**Affiliations:** Department of Neuroscience and Neurorehabilitation, Clinic Psychology Unit, Bambino Gesù Children’s Hospital, Rome, Italy; Libera Università Maria Santissima Assunta, Rome, Italy; Department of Paediatric Cardiology and Cardiac Surgery, Paediatric Arrhythmia and Syncope Unit, Bambino Gesù Children’s Hospital, Rome, Italy

**Keywords:** Pediatric syncope, Quality of life, Parenting stress, Psychosocial adjustment, Psychological symptoms, Caregiving

## Abstract

**Background:**

Health-related quality of life, which can be investigated using self-reports or parental reports, could help healthcare providers understand the subjective perception of well-being of children suffering from recurrent syncopal episodes. Quality of life is not only a measure of health but is also a reflection of patients’ and parents’ perceptions and expectations of health. This study assessed: 1) the consistency and agreement between pediatric patients’ self-reports and parents’ proxy-reports of their child’s quality of life; 2) whether this patient-parent agreement is dependent on additional demographic and clinical or distress factors; 3) whether the parents’ psychological distress influences children’s and parents’ responses to questionnaires on quality of life.

**Methods:**

One hundred and twenty-five Italian children aged 6-18 years old (Mean age 12.75, SD 2.73, 48 % female) and their parents completed the Pediatric Quality of Life inventory with self-reports and parent-proxy reports, the Parenting Stress Index - Short Form questionnaire and the Child Behavior Checklist for ages 6-18.

Patients’ and parents’ scores on quality of life were analyzed via an intra-class correlation coefficient, Spearman’s correlation coefficient, Wilcoxon signed-rank test, and Bland-Altman plot.

**Results:**

Child-rated quality of life was lower than parent-rated quality of life. However, there were no statistically significant differences between pediatric patients’ self-reports and their parents’ proxy-reports of on quality of life. Clinically significant patient-parent variation in pediatric health-related quality of life was observed. Differences in patient-parent proxy Pediatric Quality of Life inventory Total Scale Score scores were significantly associated with patient age.

**Conclusion:**

Concerning parents’ proxy-ratings of their children’s quality of life on the Pediatric Quality of Life inventory, parental stress was found to be negatively associated with their perceptions of their child’s psychological quality of life. Indeed, childhood illness is a source of stress for the whole family, and exposes family members to a greater risk of developing psychosocial difficulties. In conclusion, this study invites reflection on the use of cross-informants in investigating the quality of life of young patients with neurocardiogenic syncope and the psychological factors that influence how quality of life is perceived.

## Background

Neurocardiogenic syncope (NCS) is defined as the transient loss of postural tone and consciousness with spontaneous recovery. It is a condition that frequently occurs in childhood and adolescence [1, 2]. Although most syncopal events are benign, they are associated with a significant decrease of the quality of life by reducing the subjective perception of well-being [3] Byars and colleagues [4] studied 44 children with a history of recurrent syncope and reported adjustment difficulties, including symptoms of anxiety and social withdrawal. This result is confirmed by a longitudinal study in which children with NCS were found to be more prone to exhibit depressive symptoms than their peers in a control group [6]. European Society of Cardiology (ESC) Guidelines [3] that the cornerstone of therapy for young patients includes education and reassurance, to analyze how parents react to their children’s syndrome Effectively, parents of children and adolescents with NCS show stress related to the management of syncopal episodes, e.g., having to call the doctor, going to the emergency department, asking for help, being confused and worrying about managing the situation. Often they worry about the recurrence of the syncopal episodes, which may affect their perception of parental distress and the quality of daily life because they feel insecure and anxious, and may develop an over-controlling care attitude.

Measuring the Quality of Life (QoL) to gain a better understanding patients’ point of view is used in routine clinical trials. The measurement of QoL can be used by healthcare providers to obtain a holistic view of children’s subjective well-being in several domains of their daily life which could be informative concerning the results of treatment strategies (psychological outcomes for example) that may not be captured by traditional outcome indices. QoL studies involving pediatric patients with chronic and transitional diseases and their parents can help health practitioners to more fully understand children’s disease-specific symptoms, psychosocial functioning and development in the context of daily life [5–7]. Given the lower cognitive and language skills of young children, the majority of child QoL instruments have been developed using proxy parent reports to gain information about the children [8]. The availability of questionnaires with child and parent versions has raised questions about the level of agreement between children’s and parents’ views regarding child functioning. Some studies report a poor parent-child agreement [9], others a moderate to high agreement [10, 11]. Parent-child agreement may be affected by a number of variables related to children’s age, sex, and diseases and parents’ socio-demographic features [12, 13]. A few studies have addressed the quality of life of children with NCS. Among these, Anderson and colleagues [5] have investigated syncopal children’s subjective well-being in their daily lives compared with both healthy controls and controls with chronic illnesses. Their findings suggested that pediatric patients with syncope, although typically of benign etiology, had low health-related quality of life compared with healthy controls [14].

To the best of our knowledge, almost no studies have examined the QoL item scores to assess syncopal children’s subjective well-being compared with their parents’ estimation. The main core of the current study was to investigate NCS patients’ self-rated QoL versus their parents’ evaluation. Our first aim was to explore the degree of agreement *versus* disagreement between NCS patients’ self-reported quality of life and parents’ rating of their child’s quality of life. Secondly, we aimed to investigate how parents-children accordance *versus* discordance on child’s quality of life correlated with demographic, clinical, and psychological factors of both parents and children. The third and final goal of the present study was to explore whether parents’ proxy-reports on child’s quality of life were predicted by studied variables. In view of the lack of earlier research specifically focusing on NCS patients, we did not formulate any *a priori* hypothesis in relation to our three aims, thus this is an exploratory study.

## Method

### Sample and data collection

**Fig. 1:**
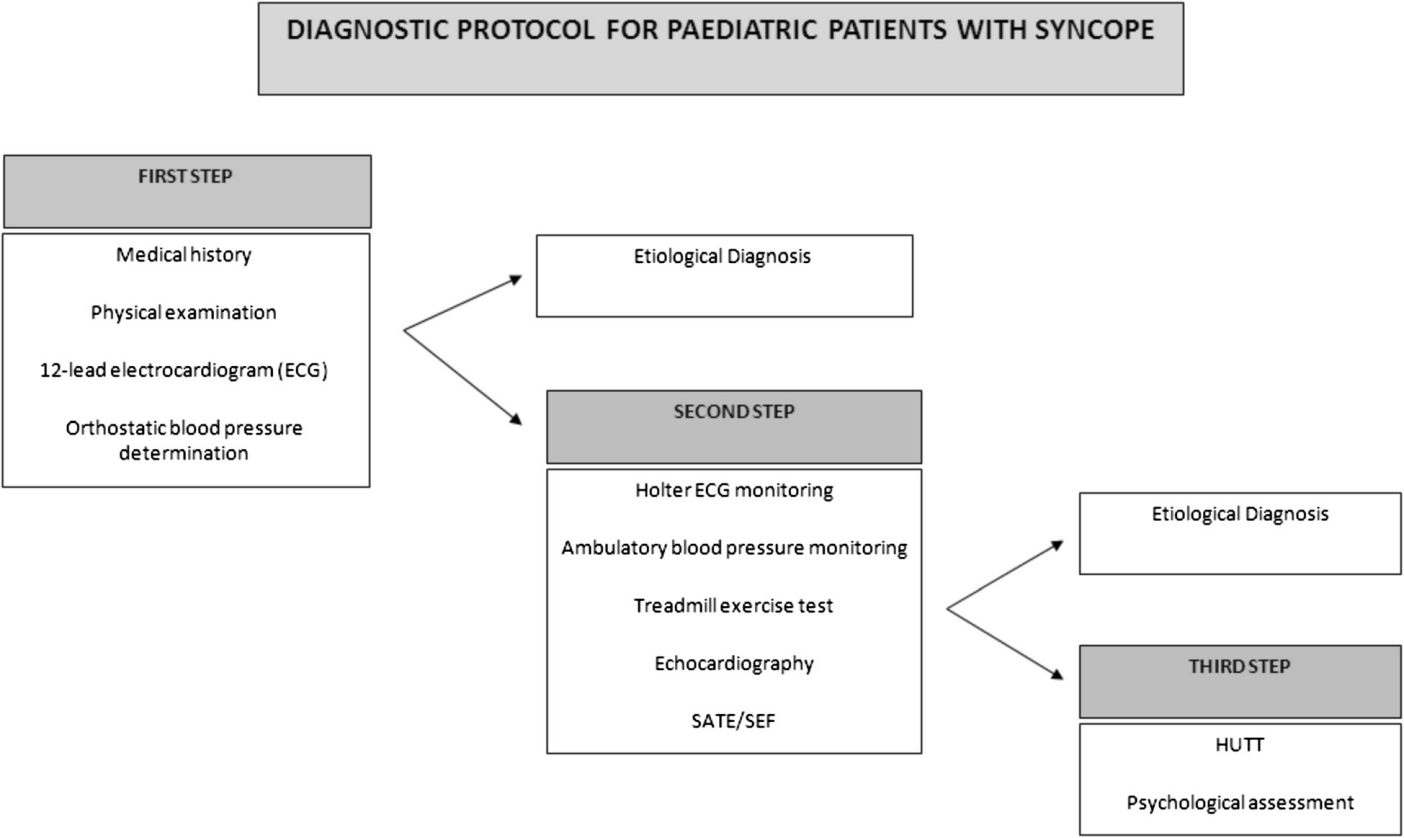
Diagnostic protocol for paediatric patients with syncope

Children and adolescents with NCS newly diagnosed between October 2011 and March 2013 at the Bambino Gesù Pediatric Hospital were eligible for this study if aged between 6 and 18 years. All patients were directly referred to the Syncope Unit of this hospital by their primary care physician or another specialist physician with a syncope diagnosis. It is estimated that ten percent of causes of death in children is represented by myocardial infarction and often the retrospective assessment reveals a history of syncope. Syncope can be classified by severity in cardiac syncope (15 %), neurocardiogenic syncope 65 %, of which 5 % Breath Holding Spells and 20 % of non-syncope (based on neurological or neuropsychiatric causes) (Fig. 1).

The patients with cognitive and mental disability and the patient don’t speak and write in Italian language were excluded. During this period we recruited 125 patients with NCS. These pediatric patients were divided into two age groups: 6.3–12.7 years old (Child group) and 12.8–18.3 years old (Adolescent group). Parents of all the participating children had given written indication of interest and signed informed consent. All procedures are in accordance with international ethical standards and were approved by the Bioethics Committee of our Institution. Patients with a diagnosis of heart, neurological or metabolic causes of syncope were excluded from this study. Patients were also excluded if the parents did not speak Italian, if the child was in foster/social care, if a mental retardation was diagnosed or the consultant felt that the parents could become unduly distressed by participating. If both parents were available, one volunteered to serve as the single study participant. All study patients had experienced at least one or two syncope episodes in the last six months.

### Study design

All the patients and their parents completed study questionnaires and health surveys at the time of their initial appointment in the Syncope Unit, but prior to being evaluated and treated by a pediatrician. The patient and parent were instructed by the study psychologist on how to complete the various measurement instruments and to complete them independently, so as to minimize any respondent cross-contamination. Patients and their parents were given ample time and privacy to complete the study forms. Parents’ demographic details, such as occupation, relationship status, ethnic origin, level of education and treatment status, are not given because not all parents gave informed consent.

### Study measurement

#### The Pediatric Quality of Life inventory version 4.0 (PedsQL™ 4.0)

The PedsQL 4.0 Generic Core Scales were used to evaluate the health-related quality of life (HR-QoL) in pediatric populations [15]. This instrument was designed to measure the core health dimensions as delineated by the World Health Organization [16], as well as school functioning. The pediatric Quality of Life Inventory (PedsQL™ 4.0, MAPI Research Institute, Lyon, France) was used in the present study, with the Italian Generic Version. PedsQL™ is a generic HR-QoL questionnaire that has both self- and parent-proxy report forms. It is a reliable and valid measurement tool of health-related quality of life in the pediatric population and is self-administrated [17, 18]. It has 23 items and four domains: physical functioning (8 items), emotional functioning (5 items), social functioning (5 items) and school functioning (5 items). It generates a total score summarizing all four domains and named the Total Health Scale, and two subscales named the Physical Health Scale, which refers to physical functioning, and the Psychosocial Health Scale which summarizes the emotional, social and school domains. A 5-point Likert-type scale is utilized (0 = never a problem; 1 = almost never a problem; 2 = sometimes a problem; 3 = often a problem; 4 = almost always a problem). Items are reverse-scored and linearly transformed to a 0 to 100 scale. Scores at or above the 75^th^ percentile represent a good perceived quality of life in both the child- and parent- forms. We used appropriate self-report versions of the PedsQL™ 4.0 for the 5–7, 8–12 and 13–18 year-olds. The parent-proxy version of the PedsQL™ 4.0 has shown adequate feasibility, reliability, and validity in parents recruited from clinical or well-child checks. The Italian Generic version of the PedsQL™ used in the present study is valid and reliable, with internal consistency for the different scales of 0.60–0.70 [19]. The reliability values were lower than those found by Varni [20], but according to Dazzi and Pedrabissi [19] can be considered adequate. The accepted minimal clinically important difference (MCID) is 4.5 points for the PedsQL™ 4.0, which is the value that was applied here [17].

#### Parent Stress Index – Short Form (PSI-SF)

The Parenting Stress Index – Short Form (PSI-SF) is a short version of a standardized tool used to assess parenting stress.

The test has 36 items (in a Likert scale of 5 points) and four domains: the Parental Distress scale (PD) that defines the level of distress which a parent experiences in his parenting role; the Parent-Child Dysfunctional Interaction (P-CDI) scale that determines a parent’s perception of a child not responding to his expectations, and of a parent-child interaction which is neither reinforcing nor rewarding; the Difficult Child (DC) scale that assesses the parent’s perception of his child as being easy/difficult to manage, based on behavioral characteristics, and finally the Total Parental Stress (TPS) scale, obtained by calculating the sum of the scores of the previous three domains. It provides both raw and percentile scores. A score at or above the 85^th^ percentile indicates high stress levels [30]. A score between the 15^th^ and 80^th^ percentile indicates a normal stress level. The PSI-SF has been widely used with comparable parent populations [21–23] and psychometric evidence supports its reliability and validity [14, 24].

#### The child behavior checklist for age 6–18 years

The risks of psychopathological features and low adaptability in children and adolescents with syncope were assessed using the standardized Italian version of the Child Behavior Checklist for ages 6–18 (CBCL/6-18) [25]. It is a parent-questionnaire with 113 problem items. Each item is scored 0 if the problem is not true for the child, 1 if the problem is somewhat or sometimes true, or 2 if it is very or often true. The checklist has three broad scales and eight syndrome subscales: Anxious/Depressed, Withdrawn/Depressed, Somatic Complaints, Social Problems, Thought Problems, Attention Problems, Rule-Breaking Behavior, and Aggressive Behavior. These subscales are useful for screening emotional and behavioral problems across multiple cultures [26] and are collected into three broad scales: a) the internalizing problems scale with 32 items (assessed according to three empirically based internalizing syndrome subscales: anxious/depressed, somatic, and withdrawal symptoms), b) the externalizing problems scale with 35 items (assessed according to two empirically based externalizing syndrome subscales: rule-breaking behavior and aggressive behavior), and c) the total problems scale. Although as yet there are no normative Italian data on the most recent CBCL, the psychometric properties of the previous CBCL version [27] have been recently investigated in a study conducted in Italy, which showed the good validity and reliability of the instrument for use in the Italian population [28]. As recommended by Achenbach, T scores were used to analyze the combined scores in the CBCL/6-18. Following the CBCL automated scoring procedure (ADM, [29]), all questionnaires with eight or more missing items were automatically discarded.

### Statistical analysis

NCS patients self-reports and parent proxy-reports on the PedsQL™ were compared using two statistical methods: 1) intra-class correlation coefficients (ICC); 2) minimal clinically important difference (MCID) [30, 31].

With the PedsQL™ three scores were obtained: Total Scale, Physical Health Summary and Psychosocial Health Summary. Taking into account children’s increasing ability to self-report their own quality of life, the agreement indexes were calculated for the total sample (*N* = 125) and separately for two patient age groups (6.3–12.7 years, *N* = 63 and 12.8–18.3 years, *N* = 62). To obtain two age groups we used the median age criteria. Secondly, using a cut-off point of 4.5 as the accepted minimal clinically important difference (MCID), the frequency distribution of patient self-reports and parent agreement was calculated for the following quality of life measures: Total Scale, Physical Health and Psychosocial Health Summary Scores.

We analyzed the zero-order correlations between the differences in the parent-child dyad reports of HRQoL and certain variables. For the patients, sex, age, recurrent syncope and CBCL internalizing and externalizing scores were taken into account. For the parents, however, for reasons of privacy no information was collected on gender and social-demographic background, but we did take into account parents’ stress index scores. The associations amongst all the above-mentioned variables were assessed using Spearman’s correlation coefficients for the total sample. A hierarchical regression model was used to evaluate a predictive model for the differences observed in the parent-child dyad reports of HRQoL. Finally, a second hierarchical regression model was used to analyze factors which affected parents-proxy reports on PedsQL™. At each step the regression equation was assessed for statistically significant variations of the coefficient of determination (*R*^*2*^) as well as beta weights [32]. Regression assumptions (e.g., homoscedasticity, multivariate normality, etc.) were checked and found to be for all variables. Finally, a *p* > 0.001 Mahalanobis’ distance criterion was used to identify and eventually skip multivariate outliers. Statistical analyses were done using IBM SPSS Statistics 22.

## Results

### Social-demographic and psychological characteristics of children with NCS and their parents

Table 1 shows demographic, clinical and psychological characteristics and descriptive variables of NCS patients. All characteristics were expressed as an average score (*M*) or in relative frequencies (%). Parents were mostly mothers (90 %), no other social-demographic characteristics were given due to privacy restrictions. Regarding parent stress scores, Parent-Child Dysfunctional Interaction obtained *M* = 23.26 (SD = 8.55), Parent Distress *M* = 26.43 (SD = 9.96), Difficult Children M = 26.22 (SD = 9.18), and Total Score Parent Stress *M* = 75.37 (SD = 22.67). The Patient sample comprised 125 subjects (48 % female) with a mean age of *M* = 12.75 (SD = 2.73). Patients were split into two groups based on their median age (*Median* = 12.7). Children aged 6.30 to 12.7 years represented 52 % of the sample (*N* = 63), while 48 % were in the 12.8 to 18.3 years group (*N* = 62). Finally, regarding their psychological factors, Externalizing problems were present in *M* = 8.76 (SD = 6.35) and Internalizing problems in *M* = 11.52 (SD = 8.09).

**Table 1:** Description of patients and parent characteristics

Number of parent–child participants	125	Normal range
*Parent*		
Sex		
Mothers	113 (90 %)	
Fathers	12 (10 %)	
Stress Index		
PD	26.43 (SD = 9.96)	26.28 (SD = 7.63)
DC	26.22 (SD = 9.18)	23.40 (SD = 7.17)
P-CDI	23.26 (SD = 8.55)	20.03 (SD = 6.11)
Total PS	75.37 (SD = 22.67)	69.70 (SD = 17.38)
*Patients*		
Mean age (*SD*)	12.75 (2.73)	
Child	63 (52 %)	
Adolescent	62 (48 %)	
Sex		
Female	48 %	
Male	52 %	
Recurrent syncope	92 (73.6 %)	
CBCL		
Externalizing	8.76 (SD = 6.35)	7.64 (SD = 5.80)
Internalizing	11.52 (SD = 8.09)	6.96 (SD = 5.53)

*SD* standard deviation; *NCS* neurocardiogenetic syndrome

Child = 6.30 to 12.70 year of age; Adolescent = 12.80 to18.30 year of age

*P-CDI* parent-child dysfunctional interaction; *PD* parental distress; *DC* difficult child; *Total PS* total parent stress

### Agreement versus disagreement between pediatric self-reported and parent proxy-reported HRQoL

#### Intra-class correlation coefficients (ICC)

Table 2 shows the intra-class correlation coefficients between patient self-reported and parent proxy-reported Total Scale Score, Physical Health, and Psychosocial Health Summary Scores on the PedsQL™. The HRQoL score consistency between study patients and their parents was significant across the entire patient age range and within the two patient age groups (child vs adolescent groups).

**Table 2:** Correlations between patients self-reported and parent reported HRQoL scores on the PedsQL

Scale	Intra-class correlation coefficients (95 % CI)		
	Total sample (*N* = 125)	Child (*N* = 63)	Adolescent (*N* = 62)
Total Score	0.63 (0.47. 0.74)	0.60 (0.34. 0.76)	0.64 (0.41. 0.76)
	*p* < 0.001	*p* < 0.001	*p* < 0.001
Physical Health Summary	0.68 (0.38. 0.64)	0.54 (0.23. 0.72)	0.79 (0.65. 0.87)
	*p* < 0.001	*p* = 0.001	*p* < 0.001
Psychosocial Health Summary	0.57 (0.38. 0.70)	0.63 (0.39. 0.78)	0.49 (0.15. 0.69)
	*p* < 0.001	*p* < 0.001	*p* = 0.005

Child = 6.3-12.7 years old

Adolescent = 12.8–18.3 years old

Parent-patient associations regarding the three measures of PedsQL™ were all statistically significant and positive. In most cases the association value was higher than 0.6 with *p* < 0.001. The lowest correlations referred to parent and younger patient dyads, particularly for the Physical Health Score. In the case of adolescent patients and their parents the lowest positive correlation was found on the Psychosocial Health Score.

#### Minimal clinically important difference (MCID)

Table 3 shows mean and standard deviation values for the patient self-reports and parent proxy-reports on PedsQL™ scores. Generally, these scores were not normally distributed and thus were subjected to nonparametric statistics the Wilcoxon test was calculated for the total sample and the two age groups of patients.

**Table 3:** Patients and Parents scores on Peds compared by means of Wilcoxon test

		Total sample (*N* = 125)	Child (*N* = 63)	Adolescent (*N* = 62)
Total Health Score Patient	*Mean (SD)*	74.71 (14.68)	73.44 (14.49)	76 (14.89)
	*Median*	76 (65.05–85.9)	76 (66.3–84.7)	78.2 (63–89)
Total Health Score Parent	*Mean (SD)*	75.89 (16.4)	74.54 (15.98)	77.26 (16.83)
	*Median (IQR)*	78.6 (66.5–89.1)	76 (66.3–88)	80 (67.48–89.38)
	*p*	0.45	0.65	0.59
Psychosocial Health Score Patient	*Mean (SD)*	75.08 (15.76)	74.69 (15.61)	75.48 (16.03)
	*Median (IQR)*	76.6 (60–88.3)	76.6 (65–88.3)	77.55 (60–90)
Psychosocial Health Score Parent	*Mean (SD)*	75.46 (17.99)	73.99 (18.36)	76.95 (17.62)
	*Median (IQR)*	80 (60–91.6)	78.3 (58.3–91.6)	81.6 (60–91.83)
	*p*	0.73	0.92	0.54
Physical Health Score Patient	*Mean (SD)*	73.38 (18.29)	70.4 (18.23)	76.83 (18.09)
	*Median (IQR)*	78.1 (60.9–87.5)	75 (59.3–84.3)	81.2 (64.83–90.6)
Physical Health Score Parent	*Mean (SD)*	77.22 (18.33)	75.56 (18.05)	78.88 (18.6)
	*Median (IQR)*	81.2 (65.6–90.6)	81.2 (65.6–87.5)	84.3 (65.693.7)
	*p*	0.027	0.39	0.34

Wilcoxon tests show no significant difference among all pairs of comparison between pediatric self-reports and parent proxy-reports on PedsQL™ scores. Despite this lack of significance the MCID values (i.e. absolute score difference of less than 4.5) indicated that parents proxy-reports on PedsQL™ scores were systematically higher than their children’s self-reported ratings for HRQoL (Table 4).

**Table 4:** Frequencies of Patient self-reported versus Parent proxy-reported PedsQL scores

	Total score	Physical health	Psycho-social health
Patient < Parent	72	56	52
Patient = Parent	0.8	19.2	17.6
Patient > Parent	27.2	24.8	30.4

Agreement defined as an absolute PedsQL score difference of less than 4.5 (MCID). Patient < Parent and Patient > Parent defined ad PedsQL scores difference of grater than or equal to 4.5

The parent-patient dyad trend for the Total Score was 72 % (patient < parent); Physical Health was 56 % (patient < parent), and Psychosocial Health Summary Score was 52 % (patient < parent).

### Predictors of patient-parent agreement about HRQoL

Zero-order correlations were calculated among the absolute differences between the patient versus parent Total Score on PedsQL™ and six independent variables (for the patients: sex, age, recurrent syncope, internalizing and externalizing problems; for the parents: stress index). None of these variables was consistently significantly associated with the study variable. More specifically, the independent variables analyzed did not predict patient-parent agreement on patient quality of life as revealed by the logistic regression model used.

### Associations between PedsQL™ and demographic, clinical and psychological factors

Table 5 shows zero-order correlations among independent variables and patients self-reports as well as parents proxy-reports on PedsQL™.

**Table 5:** Zero-Order Correlations Among all Variables

		Patients variables					Parent variables			
		Sex	Age	RS	Externalizing	Internalizing	PD	P-CDI	DC	Total PS
Patient										
	Total Health Score	-0.024	0.078	0.109	0.045	-0.123	-0.138	-0.165	-.262^**^	-.235^**^
	Physical Health	0.088	.197^*^	0.118	0.157	-0.122	-0.073	-0.034	-0.106	-0.108
	Psychosocial Health	-0.083	-0.004	0.113	-0.014	-0.069	-0.127	-.264^**^	-.334^**^	-.270^**^
Parent										
	Total Health Score	-0.109	0.135	-0.040	-0.101	-.240^**^	-.243^**^	-.227^*^	-.303^**^	-.311^**^
	Physical Health	-0.113	0.162	-0.003	-0.060	-.194^*^	-.215^*^	-0.166	-.215^*^	-.237^**^
	Psychosocial Health	-0.056	0.096	-0.059	-0.108	-.240^**^	-.216^*^	-.206^*^	-.296^**^	-.295^**^

*RS* recurrent syncope, *QLTS-P* Quality Life Total Scores (Parent), *QLPHY-P* Quality Life Physical Health (Parent), *QLPSY-P* Quality Life Psychosocial Health (Parent)

*P-CDI* parent-child dysfunctional interaction; *PD* parental distress, *DC* difficult child; *Total PS* total parent stress

*means *p* <.05; **means *p* <.001

As regards the association between patients PedsQL™ scores and independent variables, no significant correlations were found with sex, age, recurrent syncope and psychological factors for the externalizing and internalizing problems scores. With regard to patients self-reports on PedsQL™ and their parents stress indicators, a negative association was found with the Difficult Children score (*r* = -0.334, *p* < 0.01). With respect to parent proxy-reports on PedsQL™, there was no significant association with their children’s characteristics (sex, age and RS). At the same time, only internalizing problems on CBCL scores was significantly and negatively associated with the Psychosocial Health Score (*r* = -0.240, *p* < 0.01) and Total Health Score (*r* = -0.240, *p* < 0.01); however both were low associations. Furthermore, all the correlations between the three PedsQL™ scales and Parent Stress Indexes were negatively significant (*p* < 0.05 to *p* < 0.01) even with attenuated values. Finally, more interesting associations were found between the Total Health Score of parent-proxy reports and two sub-scales of their Stress Index, specifically with DC (*r* = -0.303, *p* < 0.01) and Total PS (*r* = -0.311, *p* < 0.01).

### Predicted models of parent-proxy reports on PedsQL™

Finally, a hierarchical regression model was used in the last analysis to identify which of the studied variables affected parental estimation/assessment of their child’s quality of life. Taking into account the significant correlation emerging in the above-mentioned analysis (Tables 2 and 5), *p* ≤ .01 and *r* ≥ ±.240 were used as entry criteria. More specifically, the analysis included three steps with the Total Health Score of parents on PedsQL™ as constant variable. At Step 1, patient self-reported Total Health Scores on PedsQL™ that obtained a statistically significant effect (ΔR^2^ = 0.205, F = 32.96, *p* <0.001) were entered as a single block, with Patient Total Health Score on PedsQL™ (*β* =0.460, *p* < 0.001) emerging as a significant determinant of parent-proxy reported Total Health Score. At Step 2, the Total Score of parent stress was entered as a single block. At this point a statistically significant improvement of the variation of the model was observed (ΔR^2^ = 0.23, F = 19.56, *p* <0.001) even though Total Parent Stress exerted a less significant effect (*β* = -0.182, *p* < 0.05). Finally, Internalizing problems (CBCL Internalizing) was entered at Step 3, resulting in a significant improvement of the predictive model (ΔR^2^ = 0.28, F = 16.99, *p* <0.001). At this step, it is important to highlight the decrement of the predictive effect of Total PS (*β* = -0.144, *p* = 0.70), while a significant effect to explain regression model was played by the CBCL Internalizing variable (*β* = -.236, *p* = .003).

## Discussion

The main aim of the present study was to investigate the perceived quality of life of young NCS patients by analyzing differences between child self-reports and parent proxy-reports obtained via the Peds QL™ 4.0. Our findings suggest that: 1) children and adolescents with NCS display statistically significant agreement with their parents in terms of how they perceive their overall quality of life; levels of agreement increase with age for physical health but decline with age for psychosocial wellbeing; 2) agreement between patients’ and parents’ ratings was not predicted by the clinical and psychological variables analyzed in the study (for patients: gender, age, recurrent syncope, internalizing and externalizing problems; for parents: stress index); however, there were interesting correlations between psychological factors in both patients (internalizing problems) and in their parents (stress index) and parental perceptions of QoL; 3) finally, the predictive model that best explained parents’ perceptions of the QoL enjoyed by their child comprised three factors: children’s self-reported QoL, parental stress, and internalizing problems on the part of the children.

With regard to the first of these findings, our data are in line with other studies that have found significant levels of agreement regarding QoL between parents and children suffering from a variety of pediatric illnesses [11]. They also support findings in the literature which show that, as children get older, the gap between their own self-perceptions and their parents’ views tends to narrow [13]. Nonetheless, analysis of the individual subscales that constitute the PedsQoL showed that adolescents with NCS, across all the age groups represented in our sample, rated their psychosocial QoL more poorly than did their parents. This outcome is in line with recent studies [33] on the level of agreement between child self-reports and parent proxy-reports of health-related QoL in boys with Duchenne muscular dystrophy.

However, the variable nature of the evidence gathered to date suggests that caution should be exercised when interpreting findings on parent-child agreement, which in any case should be examined in relation to other variables such as the child’s illness, age, social context, parenting style and length of time since diagnosis, given that such factors are known to impact on acceptance of the health condition and parental coping strategies [34]. Contrary to other studies [35–37] we observed a the large percentage of parents in our sample who overestimated their child’s quality of life, despite remaining significantly in agreement with their children’s self-perceptions. It is possible that these parents were responding emotionally to their child’s situation by denying the illness and its effects [37]. This aspect should not be overlooked when treating young patients whose QoL is reliant on parental care and the familial emotional atmosphere. Ideally, the parents of sick children should be trained to observe not only the more evident aspects of QoL (physical health) but also the emotional and social variables associated with physical wellbeing.

Our findings do not concord with studies that have identified a significant effect of gender on self-perceived QoL in young patients with a variety of illnesses [13, 14, 37]. The absence of correlations between the self-perceived QoL of the young NCS patients in our study and their gender, age and incidence of recurrent syncope provides key indications for future research on children and adolescents with this health condition.

Concerning parents’ proxy-ratings of children’s QoL on the PedsQoL, parental stress was found to be negatively associated with their perceptions of their child’s psychological QoL. Indeed, childhood illness is a source of stress for the whole family, and exposes family members to a greater risk of developing psychosocial difficulties [38]. A negative association was also seen between young patients’ internalizing problems scores and their parents’ proxy-reports of psychosocial QoL. This result would seem to be in line with Varni’s biobehavioral model as well as with Palermo and Chambers’ integrative model of pediatric chronic pain [39].

In general, our data suggest that pediatric patients with syncope, despite the typically benign etiology of this condition, are at greater risk when it comes to psychological health, whether assessed using self-report (e.g. self-rated psychosocial QoL) or adult-report (e.g. internalizing scores) measures. This result is confirmed by a longitudinal study in which children with NCS were found to be more prone to exhibit depressive symptoms than their peers in a control group [6]. Nonetheless, follow-up research with healthy controls is recommended to further consolidate this finding.

The third and final result of the current study concerns the testing of a predictive model showed that the following variables have a progressively predictive effect on parent-proxy-ratings of patients’ QoL: children’s self-reported QoL, parental stress and, finally, patients’ internalizing issues. In sum, the quality of life that parents attribute to their children with NCS is predicted not only by variables linked to the young patients themselves, such as self-reported QoL and internalizing problems, but also by parental stress. This may be due to the fact that the three subscales of the parent stress index– dysfunctional parent-child interaction, parental distress, and difficult child – all assess aspects of how parents perceive and experience their child’s condition. It would seem that parental attribution of satisfactory QoL, despite the physical conditions associated with NCS, depends on how the parents perceive their own relationship with their difficult child.

## Conclusion

In conclusion, this study invites reflection on the use of cross-informants in investigating the QoL of young patients with NCS. Since the assessment of chronically ill children and adolescents can also facilitate improvements in clinical decision making, evaluation of the quality of medical care, estimation of the health care needs, and an understanding of the causes and consequences of differences in health [40], the treatment of pediatric NCS patients should include information on these psychological domains both from the children and from their parents. Furthermore, to optimize the management of chronic illness in these young patients, as well as being familiar with the different ways in which a state of ill-health may be perceived, it is important to foster cross-communication amongst all those involved. In light of the key role played by parents in the care and treatment of a sick child, it is vital to attend to patents’ mental health and their perception of their own lives and that of their child.

## Abbreviations

HRQoL: Health-related quality of life
QoL: Quality of Life
CBCL: Child behavior checklist
PSI: Parenting stress index
Peds QL™ 4.0: Pediatric quality of life inventory version 4.0
NCS: Neurocardiogenic syncope
